# Trabecular Bone Component Assessment under Orthodontic Loads and Movements during Periodontal Breakdown—A Finite Elements Analysis

**DOI:** 10.3390/dj12060190

**Published:** 2024-06-20

**Authors:** Radu-Andrei Moga, Cristian Doru Olteanu, Ada Gabriela Delean

**Affiliations:** 1 Department of Cariology, Endodontics and Oral Pathology, School of Dental Medicine, University of Medicine and Pharmacy Iuliu Hatieganu, Strada Motilor 33, 400001 Cluj-Napoca, Romania; ada.delean@umfcluj.ro; 2 Department of Orthodontics, School of Dental Medicine, University of Medicine and Pharmacy Iuliu Hatieganu, Strada Avram Iancu 31, 400083 Cluj-Napoca, Romania

**Keywords:** trabecular bone, failure criteria, periodontal breakdown, light orthodontic force, finite elements analysis, orthodontic movements

## Abstract

This numerical analysis, by employing Tresca and Von Mises failure criteria, assessed the biomechanical behavior of a trabecular bone component subjected to 0.6, 1.2, and 2.4 N orthodontic forces under five movements (intrusion, extrusion, tipping, rotation, and translation) and during a gradual horizontal periodontal breakdown (0–8 mm). Additionally, they assessed the changes produced by bone loss, and the ischemic and resorptive risks. The analysis employed eighty-one models of nine patients in 405 simulations. Both failure criteria showed similar qualitative results, with Tresca being quantitatively higher by 1.09–1.21. No qualitative differences were seen between the three orthodontic loads. Quantitatively, a doubling (1.2 N) and quadrupling (2.4 N) were visible when compared to 0.6 N. Rotation and translation followed by tipping are the most stressful, especially for a reduced periodontium, prone to higher ischemic and resorptive risks. In an intact periodontium, 1.2 N can be safely applied but only in a reduced periodontium for extrusion and intrusion. More than 0.6 N is prone to increasing ischemic and resorptive risks for the other three movements. In an intact periodontium, stress spreads in the entire trabecular structure. In a reduced periodontium, stress concentrates (after a 4 mm loss—marker for the stress change distribution) and increases around the cervical third of the remaining alveolar socket.

## 1. Introduction

The role of the mandibular bone is for support and protection while its internal micro-architecture is important when performing several types of orthodontic treatments [1,2,3]. If intact bone assures the best force transfer (i.e., absorption and dissipation of loads and stresses), once the periodontal breakdown progresses, the height reduction fundamentally changes bone biomechanics [1,2,3]. There is little knowledge available about bone loss biomechanics in the current research literature [1,2].

There are finite element (FEA) studies that have assessed the implant–bone (most [4,5,6,7,8,9,10,11,12]) and tooth–bone (only a few [13,14,15]) stress distributions only in the intact periodontium under one or two applied loads and forces, reporting variable results that sometimes contradict clinical knowledge [4,5,6,7,8,9,10,11,12,13,14,15,16]. However, no studies individually investigating the two bone components (cortical and trabecular) were found despite the importance of knowing the individual biomechanical behavior of each bone component, especially if various levels of bone loss are present.

For preserving the remaining bone during orthodontic treatment, it is important to understand how bone reacts under forces and where their highest concentrations are, as well as the correlations among them, since periodontal disease is currently present in both younger and older patients [2,3,4,17].

From a biomechanical point of view, the bone internal micro-architecture allows the elastic deformation of its internal geometry, providing a strong structure with minimum tissular volume [1,3,4,17,18,19]. The cortical component is highly mineralized and compact, with the main roles of support and protection [1,3,4,17,18]. The trabecular component (inside cortical component) holds bone marrow, with circulatory vessels and nerves with regenerative, nutrition, and vascularization functions [1,17,18,20]. Nevertheless, along with the progression of bone loss, the bone biomechanics changes, and if the same amount of force safely applied in intact bone is kept, ischemic and resorptive risks are expected, resulting not only in further tissular loss but also alterations in the orthodontic treatment prognosis [1,17,20].

The only method available for this type of assessment of stress distribution is FEA, which enables the individual study of each tissular component under several types of conditions and forces [2,4,5,6,11,16,19,21,22,23,24]. Despite being widely used in dentistry in the past two decades, numerical studies reported various contradictory results that were sometimes debatable, often in disagreement with clinical data [1,2,17,23]. However, in the engineering field, the same method was successfully employed, renowned for its accuracy [1,2,17,20,22,23]. These results are due to the misunderstanding of FEA requirements (i.e., proper failure criteria employment, boundary condition assumptions, and anatomically correct 3D models) [1,17,22,23].

The earlier dental FEA studies [4,5,6,7,8,9,10,11,12,13,14,15,24] used several types of failure criteria (without any scientific reasoning), anatomically inaccurate models, and no correlation with physiological constants [i.e., maximum hydrostatic pressure (MHP) of 16–22 KPa (about 80% of the systolic pressure) [23] that, if exceeded, induces ischemia and further bone loss]. Among these, only three older bone–tooth reports were found to assess bone stress distribution in an intact periodontium (0.35–0.5 N of tipping [13,14]; 10 N of intrusion; 3 N of tipping and translation [15], using Von Mises and maximum principal stress failure criteria, with no correlation with the maximum hydrostatic pressure and type of analyzed material), reporting variable results that contradicted clinical data.

However, earlier FEA biomechanical studies of our group reported that correct results can be achieved in dental numerical studies, if the FEA method requirements are closely followed [1,17]. Thus, the employed failure criteria should be Von Mises (VM—homogenous) and/or Tresca (T—non-homogenous), both appropriate for the ductile-like dental tissues (with a certain brittle flow mode), while the anatomically correct 3D models should be based on CBCT (cone-beam computed tomography) records of at least 0.075 mm in voxel size [1,2,17,20,22,23]. Moreover, the assumptions of linear elasticity, isotropy, and homogeneity/non-homogeneity are appliable in up to 2.4 N (240 gf) of orthodontic loads [1,17]. Nevertheless, it must be emphasized that as FEA is a numerical study, it needs to be correlated with clinical data and physiological constants since it cannot accurately reproduce clinical conditions [24].

Besides the absorption and dissipation of stresses (due to structural elastic deformation), if the applied loads exceed structural resistance, both trabecular and cortical bone suffer from internal microcracks and damages (i.e., microscopic linear and diffuse microfractures and microcracks that quickly heal) [25]. Microfractures and microcracks were reported to be present near resorption sites, with a strong influence over the local biomechanics [25]. Age changes the internal biomechanical response to stress distribution, since bone loses some of its elasticity, becoming more brittle (older people are more sensitive to compressive stresses with microcracks, while in younger persons, bone is more ductile (more sensitive to tensile stresses)) [25]. Older people are more prone to micro-fractures of the trabecular bone when subjected to shear stresses, with a mix of brittle–ductile flow-mode biomechanical behaviors [25].

The brittle material usually does not deform and, when loads are applied, has the tendency to fissure and crack [1,2,17]. The ductile materials deform elastically under loads and recover their original form when the loads are removed [1,2,17]. The bone seems to behave like a ductile-resembling material but with a certain brittle flow mode (influenced by age, as mentioned above) [1,17]. From an internal micro-architectural point of view, the bone as a continuum is an anisotropic, non-homogenous, and anisotropic material. It must be emphasized that the biomechanical behavior of bone is multifactorial, being influenced not only by the bone continuum but also by material and physical properties [26].

When the orthodontic loads are applied, most of the stresses are absorbed and dissipated by the periodontal ligament (PDL), while the rest are transmitted to the bone, producing recoverable elastic deformations [1,17,20,22]. However, when the PDL suffers from progressive reduction during periodontal breakdown, a higher amount of stress is induced at the bone level (the trabecular bone is more sensitive to ischemia with a further loss due to rich vascularization), with ischemia, microfracture/microcracks, and bone resorption [1,17,20]. In physical biomechanics, when materials with different elastic moduli interact (Table 1, the higher the modulus, the smaller the deformation [6,21]), the highest stress appears at the contact point (cervical third of the bone) [1,17,22].

Most bone–implant FEA studies employed VM criteria in an intact periodontium, showing the stress concentration area in the cortical cervical third of the implant socket, with a wider area in the trabecular bone due to its higher ductility [4,5,6,7,8,9,10,11,15], reporting shear stress as responsible for implant socket resorption [5]. For reducing ischemic and resorptive risks, exceeding the MHP should be avoided especially in the PDL and neuro-vascular bundles. Nevertheless, trabecular bone is less deformable and vascularized than earlier tissues and can biomechanically withstand higher stresses without suffering any risks. Orthodontic movement is triggered by the circulatory disturbances in both the PDL and bone (trabecular bone due to vascularization), inducing bone remodeling [19,27,28,29]. However, if there is bone loss present or if disturbances persist for a longer period, ischemia will lead to further resorption. The clinically safely applied orthodontic forces reach up to 1.5 N (150 gf) [1,2,17,30] for an intact periodontium; nevertheless, there is no consensus about this issue. Moreover, there are no data about the safely applied force during periodontal breakdown.

An issue that can also arise during orthodontic treatment related to ischemic and resorptive risks is external orthodontic root resorption [31,32,33,34,35,36,37,38,39,40]. Orthodontic root resorption (both internal [41] and external [2]) is a side-effect that is difficult to predict [33,34,35] and is of interest in the dentine and cementum in both the root (apical and cervical) [2,41,42] and tooth crown [33,36,37]. The biomechanical process taking place is not yet entirely understood [43,44,45,46,47], resulting in small lacunae appearing after 10–35 days of applied continuous force [33,37,38,39]. The force magnitude considered safe is cited to be 0.5–1 N (for an intact periodontium) [2,41]; however, there are reports of a larger interval of 0.28–3.31 N [19,24,48,49,50,51,52], thus still being a subject of debate. There are no identified orthodontic root resorption studies for the reduced periodontium except for our earlier one [2,41]. Root resorption could also be induced by other associated factors such as age, population, osteoporosis, congenital syndromes, earlier trauma, or even endodontic treatments [31,32,40,42,49,53,54,55,56,57,58,59].

Before studying trabecular bone at the micro-level (bone cells and their interactions), a clear image of stress distribution areas in the entire structure (macro-level approach) must be obtained. We must emphasize that we found no studies about the stress distribution in trabecular bone during periodontal breakdown (how bone loss alters the stress distribution, or how/if the applied orthodontic force is recommended to be reduced), with our study being the first of its kind.

Thus, our aims were (a) to biomechanically assess the trabecular bone under small orthodontic loads, as well as five movements during a horizontal periodontal breakdown; (b) to evaluate the changes in stress distribution produced in trabecular bone by the bone loss; and (c) to assess ischemic and resorptive risks by correlations with MHP, other FEA reports, and available clinical data.

## 2. Materials and Methods

This study is part of a larger stepwise research study (clinical protocol 158/02.04.2018), numerically studying the biomechanical behavior of dental tissues under orthodontic loads and movements during periodontal breakdown.

***Patient selection.*** Herein, research with a focus on trabecular bone was conducted using eighty-one 3D models of the second lower premolar, with a total of 405 finite element simulations. The sample size was nine (9 patients, 4 males/5 females, mean age 29.81 ± 1.45) as in our previous studies [1,2,17,20,22,23], nine times larger than current FEA studies [4,5,6,7,8,9,10,11,12,13,14,15,21,27,28] that used a sample size of one (one patient/one model, few simulations). This sample size of one is specific to finite element analyses because of the strong possibility to vary experimental conditions, leading to different results.

The inclusion criteria for the region of interest were as follows: a complete mandibular dental arch, no malposition, intact teeth (no endodontic treatment/dental filling/crown), no advanced bone loss, a non-inflamed periodontium, orthodontic treatment indication, and proper oral hygiene. The exclusion criteria were as follows: a less common root geometry (e.g., non-fused double root, angulated root, extreme curvature), abnormal crown shape, deciduous teeth, abnormal root surface defects (e.g., external root resorption) or bone shape (various types of radiologically visible bone defects), abnormal pulp chamber (internal resorption), more than 2–3 mm of bone loss, and any signs of an inflamed periodontium or bad oral hygiene after acceptance.

The region of interest was the two lower molars and premolars and was recorded using X-rays with a CBCT (ProMax 3DS, Planmeca, Helsinki, Finland) with a voxel size of 0.075 mm. The lower mandibular region was selected since most of the earlier FEA studies analyzed molar and incisor regions, and little data were available related to this region.

**Figure 1:**
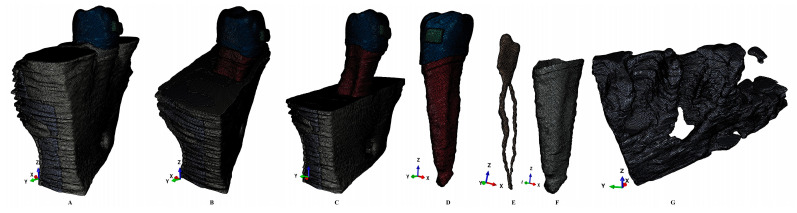
Mesh model: (**A**) second lower-right premolar model with intact periodontium, (**B**) 4 mm of bone loss, (**C**) 8 mm of bone loss, (**D**) second lower premolar, (**E**) dental pulp, (**F**) intact PDL, (**G**) trabecular bone structure in intact periodontium.

***Model creation.*** The DICOM images of various grey shades were imported into Amira 5.4.0 (Visage Imaging Inc., Andover, MA, USA) reconstruction software. However, because of the extreme tissular complexity and for anatomical accuracy, the manual reconstruction was performed by a single skilled clinician, to avoid any potential interpretation errors. Thus, enamel, dentine, dental pulp, the neuro-vascular bundle (NVB), the periodontal ligament, and trabecular and cortical bone components were identified and segmented (Figure 1). It was not possible to separate the cementum from the dentine, and, due to similar physical properties (Table 1), it was reconstructed as dentine. The periodontal ligament had a variable thickness of 0.15–0.225 mm and included the NVB. All above-mentioned tissular components were assembled into a 3D model, obtaining nine models with a second lower premolar and various levels of bone loss limited to the cervical third. The other teeth were not reconstructed, while their alveolar sockets were filled with trabecular and cortical bone. The base of a stainless steel bracket was reconstructed on the vestibular side of the premolar crown (to avoid any potential influence related to the slot of the bracket). The missing bone and PDL were manually reconstructed as close as possible to anatomical reality, thus obtaining nine models with an intact periodontium.

Each of the nine intact periodontium 3D models was subjected to a gradual horizontal periodontal breakdown of 1 mm from 0–8 mm of loss, thus obtaining nine models with various levels of bone loss from each intact periodontium model, totaling eighty-one models.

**Figure 2:**
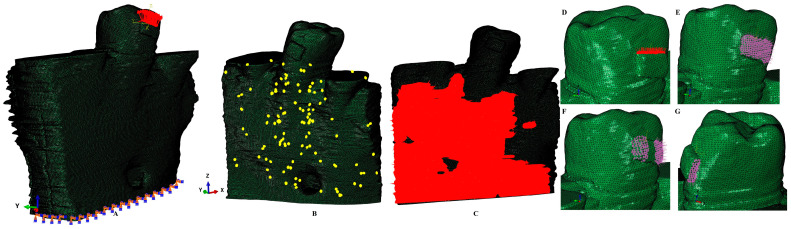
Boundary conditions: (**A**) applied extrusion vectors, (**B**) element warnings in trabecular bone component, (**C**) intact periodontium trabecular cone component, (**D**) intrusion vectors, (**E**) tipping vectors, (**F**) rotation vectors, (**G**) translation vectors.

The intact periodontium models had 5.06–6.05 million C3D4 tetrahedral elements, 0.97–1.07 million nodes, and a global element size of 0.08–0.116 mm (Figure 1 and Figure 2).

Since the manual reconstruction process, the surface of the models displayed a limited number of element warnings but no element errors (Figure 2). These element warnings are surface anomalies displayed in the non-essential regions, while there is a quasi-continuity in the stress areas. All models passed the internal checking algorithms, thus dropping any potential problems. The model with the highest number of elements displayed a total of 264 element warnings (0.0043% from the 6.05 million elements), from whom only 70 element warnings (0.0041% from the 1,699,730 elements) were displayed by the trabecular bone (Figure 2).

***FEA analysis.*** The numerical analysis was performed in Abaqus 6.13-1 (Dassault Systèmes Simulia Corp., Maastricht, The Netherlands) software, simulating five of the most common orthodontic movements (intrusion, extrusion, translation, rotation, and tipping) under three applied orthodontic loads: 0.6 N (approx. 60 gf); 1.2 N (approx. 120 gf); and 2.4 N (approx. 240 gf). The loads were applied at the bracket level. The base of the model had zero displacements (encastered), while all components were assumed to be perfectly bonded. The amounts of loads were selected since they are relatively safely applied in an intact periodontium. The homogeneity/non-homogeneity, isotropy, and linear elasticity were assumed, like most of the FEA analysis mentioned above [4,5,6,7,8,9,10,11,12,13,14,15,21,27,28], and were based on the results of an earlier study by our team that reported the correctness of these assumptions up to a load of 2.4 N [1,17].

The failure criteria employed were the ductile materials with Von Mises (overall stress, homogenous) and Tresca (shear stress, non-homogenous). In the results, the projections of stress area (red–orange, high; yellow–green, moderate; blue, low) were color-coded and accompanied by the quantitative stresses for each of the eighty-one models. The stress was evaluated for each of the three thirds of the alveolar socket. These data were then correlated with the 16–22 KPa of MHP, FEA reports, and clinical data for assessing both their correctness and ischemic and resorptive risks.

## 3. Results

**Figure 3:**
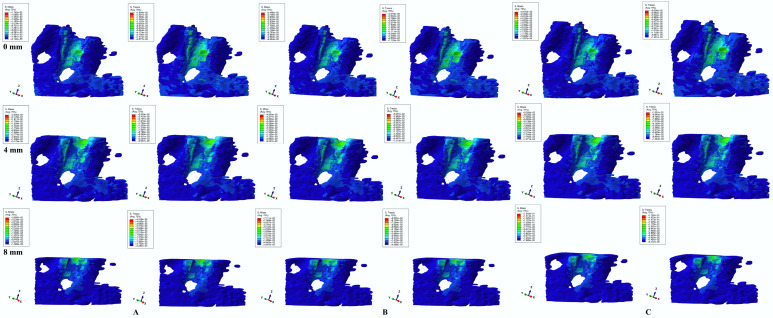
Comparative stress display of two of five failure criteria (Von Mises and Tresca) in intact, 4, and 8 mm periodontal breakdown for the intrusion movement under 0.6 (**A**), 1.2 (**B**), and 2.4 N (**C**).

**Figure 4:**
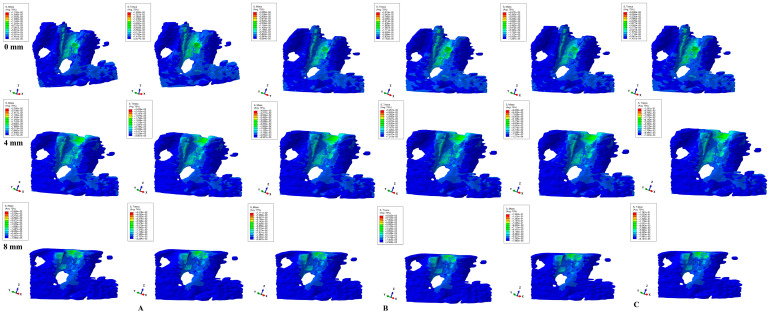
Comparative stress display of two of five failure criteria (Von Mises and Tresca) in intact, 4, and 8 mm periodontal breakdown for the extrusion movement under 0.6 (**A**), 1.2 (**B**), and 2.4 N (**C**).

**Figure 5:**
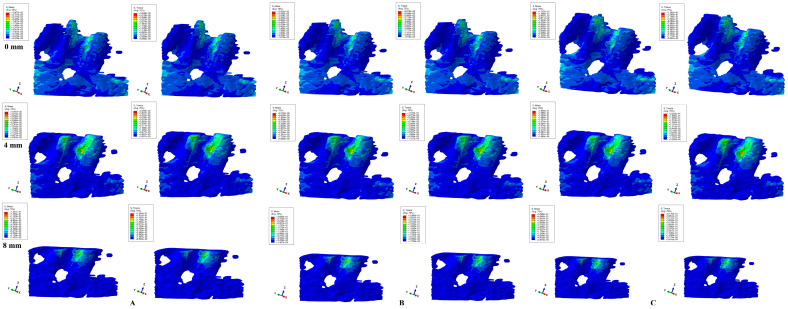
Comparative stress display of two of five failure criteria (Von Mises and Tresca) in intact, 4, and 8 mm periodontal breakdown for the translation movement under 0.6 (**A**), 1.2 (**B**), and 2.4 N (**C**).

**Figure 6:**
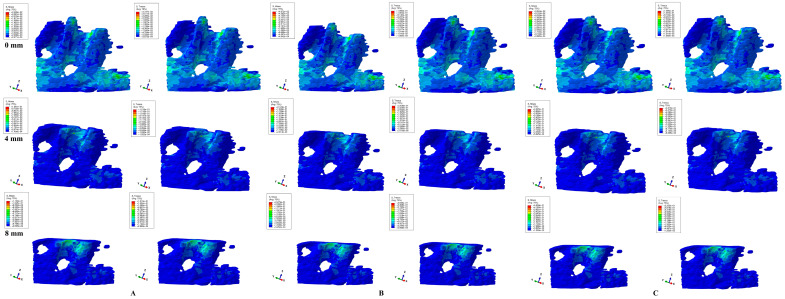
Comparative stress display of two of five failure criteria (Von Mises and Tresca) in intact, 4, and 8 mm periodontal breakdown for the rotation movement under 0.6 (**A**), 1.2 (**B**), and 2.4 N (**C**).

**Figure 7:**
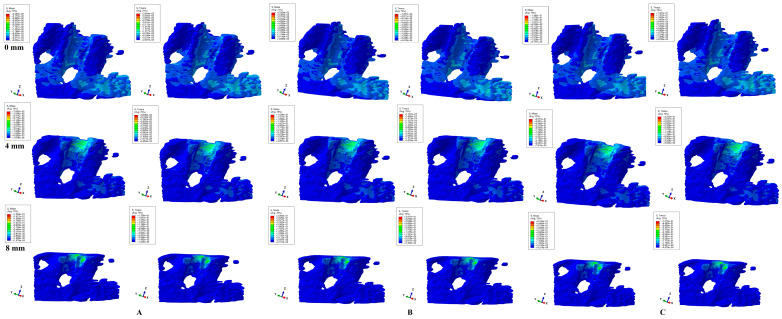
Comparative stress display of two of five failure criteria (Von Mises and Tresca) in intact, 4, and 8 mm periodontal breakdown for the tipping movement under 0.6 (**A**), 1.2 (**B**), and 2.4 N (**C**).

Herein, in our numerical analysis, there were a total of 405 simulations of eighty-one mandibular 3D models (Figure 3, Figure 4, Figure 5, Figure 6 and Figure 7 and Table 2), showing qualitative and quantitative results.

Quantitatively (Table 2), both studied failure criteria showed the highest amount of stress for rotational and translational movements, followed by tipping, while the least stresses were shown by intrusion and extrusion. Thus, rotation and translation seemed to be the most stressful movements for the trabecular bone among the five studied.

In the intact periodontium, loads of 0.6 N showed quantitative stresses not exceeding the MHP. A force of 1.2 N was safely applied for intrusion, extrusion, and tipping, while for rotation and translation, the MHP was exceeded slightly in the middle and cervical thirds. A force of 2.4 N seems to have been safely applied only for intrusion, extrusion, and tipping.

In the reduced periodontium, 0.6 N showed quantitative stresses under MHP with up to 8 mm of bone loss for intrusion and extrusion and up to 4 mm of loss for the rest of the movements. Between 4 and 8 mm of loss, a load of 0.6 N showed amounts of stress exceeding the MHP in the middle (3 times, rotation, 8 mm of loss) and cervical thirds (6 times, rotation, 8 mm of loss), suggesting high ischemic and resorptive risks. An extrusion and intrusion of 1.2 N could be relatively safely applied up to 8 mm of loss (a doubling/tripling of the alveolar socket cervical third stress for 8 mm of loss). The same amount exceeded the MHP for rotation, translation, and tipping after the first millimeters of bone loss, suggesting higher ischemic and resorptive risks. A load of 2.4 N significantly exceeded the MHP for all five movements, seeming to have significant ischemic and resorptive risks (Table 2).

Quantitatively, T criteria showed amounts of stress higher (intact periodontium: 1.09–1.21 times higher; reduced periodontium: 1.13–1.16 times higher) than those of VM, following the 1.15–1.30 times higher maximum range specified in the literature. Both criteria showed the constant expected increase pattern during the entire periodontal breakdown process following the acknowledged clinical biomechanical behavior. All quantitative stresses were lower than the acknowledged trabecular bone physical limits (compressive modulus of 0.155 GPa; compressive strength of 6 MPa [4,5,6,7,8,9,10,11,12]).

Qualitatively, both failure criteria showed similar stress areas for all five movements and bone loss levels, in line with known clinical reality. Qualitatively, there were no differences between 0.6, 1.2, and 2.4 N load stress areas, but only quantitative differences (doubling for 1.2 N and quadrupling for 2.4 N when compared with 0.6 N), showing that only the amount of stress changed when increased loads were applied, while there was a constancy of stress areas. Moreover, if, in the intact periodontium, the applied loads produced stresses in the entire trabecular bone structure, as the periodontal breakdown progresses (especially after 4 mm of loss), the displayed stresses would tend to concentrate around the remaining bone alveolar socket, but with increased quantitative amounts.

Intrusion and extrusion movements (Figure 3 and Figure 4) showed similar qualitative and quantitative results for all bone loss levels and applied loads. In the intact periodontium, the color-coded stress areas are mostly green shades (moderate, with negligible ischemic risk), found mainly in the cervical and middle third around the alveolar bone socket (lingual and proximal sides—in correspondence with the direction of force application), but with various blue shades spread in the entire cortical bone structure. As the bone and periodontal ligament loss progresses, stress tends to concentrate more in the cervical third (lingual, mesial, and distal sides) of the remaining alveolar socket (visible after 4 mm of loss), but with yellow-green areas (with a higher ischemic risk than in no bone loss).

Translational movements (Figure 5) showed a nearly similar color-coded projection for both criteria. If in the intact periodontium, the displayed stress (green shades, moderate ischemic risk) involves the entire alveolar socket (mostly proximally in agreement with the applied load direction), after 4 mm of loss, red-orange (high ischemic risk) areas are visible in the proximal (mesial and distal) alveolar socket cervical third. The translational movement in the intact periodontium, despite showing low stress (green), has a larger extension (blue-green shades) to the entire trabecular bone, while as bone loss progresses (especially after 4 mm of loss), the stress areas tend to concentrate (red-orange and yellow-green) around the alveolar socket (mostly in cervical third).

Rotation movement (Figure 6) showed similar stress areas for both analyzed criteria. In the intact periodontium, the movement showed the widest spread of stress among the five movements, not only around the alveolar socket (lingual, mesial, and distal) but also in the entire trabecular structure, and with the same green shades for the stress color-coded projections. In the reduced periodontium, the same tendency (seen in the other movements) of stress concentration and increase (yellow-green) around the remaining resorbed alveolar socket (in cervical third) is visible after 4 mm of loss.

Tipping (Figure 7) showed, in the intact periodontium, blue-green stress that was spread in the entire trabecular structure, with the stress area concentration in the middle and apical third of the alveolar socket (mesial, distal, and lingual). After 4 mm of loss, there is a visible stress concentration and increase (yellow-green) in the cervical third of the remaining alveolar socket. All three loads and both criteria showed similar quantitative results.

## 4. Discussion

In this study, a numerical investigation (405 FEA simulations on 81 3D models) assessed the biomechanical behavior of the trabecular bone structure in a gradual horizontal periodontal breakdown under five orthodontic movements and three orthodontic loads. We must emphasize that this is the first study of this type, and no other FEA studies investigating trabecular components were found in the current research literature. Moreover, no data are available about the safely applied force for periodontal breakdown (except ours [1,2,17,20,22,23]), despite reports (still debatable) of up to 1.5 N being considered safely applied in an intact periodontium [16,24,30].

The scientifical importance of our results proves that FEAs can deliver correct biomechanical results in accordance with clinical reality, since none of the previously available numerical studies [4,5,6,7,8,9,10,11,12,13,14,15] (except ours [1,2,17,20,22]) biomechanically assessed the clinical correctness of their results or followed FEA study requirements [4,5,6,11,21]. The clinical significance resides in the complete biomechanical behavioral picture of orthodontic movements in a gradual horizontal breakdown when increasing the orthodontic loads (also the first study to address this clinically critical issue).

To obtain correct results, our FEA closely followed requirements of numerical studies (i.e., failure criteria based on the type of analyzed material, boundary condition assumptions, and anatomically correct 3D model) [1,2,17,19,20,21,22]. The failure criteria biomechanically describing the ductile material behavior (resembling dental components [1,2,17]) were Von Mises (overall stress—homogenous) and Tresca (shear stress—non-homogenous), with a reported difference of 1.15–1.30 (average in this study of 1.09–1.21, falling into this range). The biomechanical assumptions of boundary conditions were isotropy, linear elasticity, and non-homogeneity/homogeneity (as in previously available FEA studies [4,5,6,7,8,9,10,11,12,13,14,15]). Correlations with physiological data were made with an MHP of 16–22 KPa and accepted clinical orthodontic biomechanical behavior [1,2,17,19,20,22].

Since both criteria show the same color-coded projections, it seems that there is a slight difference between the assumed trabecular bone homogeneity (VM) and non-homogeneity (T), with both criteria similarly describing the correct biomechanical behavior, in agreement with clinical data and earlier reports [1,17]. Moreover, it seems that these above-mentioned boundary assumptions are correct up to 2.4 N in the study of trabecular bone. Thus, an overlap between overall stress and maximum shear stress when qualitatively describing the biomechanical behavior of trabecular bone was visible. The same overlap was quantitatively visible during all five movements, for bone loss levels and loads, with T being on average 1.09–1.21 times higher than VM (of importance for ischemic and resorptive risk assessments especially in situations where MHP is exceeded).

All color-coded projections of stress distribution areas (Figure 3, Figure 4, Figure 5, Figure 6 and Figure 7) biomechanically correctly described the orthodontic movements, in agreement with acknowledged clinical behavior. Rotation seemed to be the most stressful movement, closely followed by translation, especially for a reduced periodontium where ischemic and resorptive risks increased along with bone loss progress (while intrusion and extrusion were the least) in concordance with our earlier reports [1,2,17].

If, in an intact periodontium with up to 1.2 N of load, there seems to be no major ischemic and resorptive risks, in a reduced periodontium, 1.2 N could be used only for extrusion and intrusion (with care after 7 mm of loss). For the other three movements, more than 0.6 N is prone to increasing ischemic and resorptive risks; thus, a reduction in applied loads should be considered (in agreement with earlier reports of 0.2–0.4 N for a reduced periodontium to avoid any risks [1,17] and Proffit et al. [30] of 0.1–1 N). Nevertheless, the weakest component in the periodontium is the periodontal ligament (PDL) and neuro-vascular bundle (NVB), which are highly influenced by the applied orthodontic loads (with primarily absorption and dissipation functions [1,17]), while trabecular bone is more resilient. Thus, the applied orthodontic loads in the reduced periodontium should consider PDL-NVB as more sensible rather than trabecular bone [1,3,17,18,23,25,26]. Moreover, in clinical biomechanics, there are no pure movements (as shown in simulations in this study). Thus, in clinical reality, there is a variable combination of stress distribution areas (with figures creating a general complete picture) and with amounts lower than those in this study. In an intact and especially reduced periodontium, 2.4 N should be carefully considered since it highly exceeds the MHP (e.g., up to 29 times for 8 mm of loss in rotational movement), leading to increased ischemic and resorptive risks (in agreement with other reports [1,17]).

Among the ischemic and resorptive risks, internal and external orthodontic root resorption [2,41] is another side-effect of exceeding the MHP, along with further periodontal loss [31,32,33,34,35,36,37,38,39,40]. Despite the mechano-biology behavior not being entirely understood [43,44,45,46,47], for an intact periodontium, there are reports of a safe applied force interval of 0.5–1 N as well as a larger interval of 0.28–3.31 N [19,24,48,49,50,51,52]. It must be emphasized that the external root resorption can also be induced by a multitude of other associated factors [31,32,40,42,49,53,54,55,56,57,58,59]. However, the subject in this study is the biomechanical behavior of orthodontic force in both an intact and reduced periodontium; thus, the discussion favors only orthodontic root resorption under ischemic conditions. Most orthodontic root resorption studies are limited to an intact periodontium, as well as the above-mentioned applied force interval. There are no studies investigating the orthodontic internal and external resorption in a reduced periodontium except our previous studies [2,41]. These two FEA studies (with the same method as ours, and an applied force of 0.6 and 1.2 N) reported rotation and translation prone to a higher risk of external root resorption after 4 mm of loss and resorptive risks increasing along with the progression of periodontal breakdown if the same amount of applied force is guarded [2,41]. Moreover, the internal resorptive risks are less than the external ones, increasing with the progression of periodontal breakdown, especially after 4 mm [2,41]. The internal and external surface high-stress areas are strictly correlated, while rotation and tipping showed the highest resorptive risks for the pulp chamber, decreasing with the bone loss [2,41]. The conclusion of both numerical studies was that resorptive risks increase along with the progression of periodontal breakdown if the same applied force is kept [2,41]. These reports agree with the above-mentioned findings of this study.

Biomechanically, in the intact periodontium (due to the absorption–dissipation role of the PDL-NVB intact surface), the stress is spread in the alveolar socket and entire trabecular structure. In the reduced periodontium, there is a visible concentration and increase in stress around the cervical third of the remaining alveolar socket after 4 mm of loss for all orthodontic movements and loads due to changes in stress distribution produced by the reduction in periodontal ligament surface. Thus, it seems that 4 mm of loss is a marker for the change in stress distribution during periodontal breakdown, in agreement with our earlier reports [1,17]. Overall, a visible correlation between bone loss progress, changes in stress distribution (especially concentrations around the alveolar socket after 4 mm), and quantitative stress increase (with MHP being exceeded) was seen (similar with other reports [1,17,23]).

Multiple studies on the FEA of intact periodontium bone implants [4,5,6,7,8,9,10,11,12] are available (i.e., cortical component surrounding the implant), employing the same failure criteria (VM), boundary assumptions (isotropy, homogeneity, and linear elasticity), and a sample size of one (one patient, one model, and few simulations), reporting qualitative stress concentrations around the implant alveolar socket cervical third. However, since these simulations did not include PDL-NVB components, further correlations are impossible.

Only three intact periodontium bone–tooth numerical studies were found [13,14,15], while no reduced periodontium analyses were available. These studies [13,14,15] employed the same boundary assumption and failure criteria as our study, with a sample size of one.

Shaw et al. [13] (sample size of one, upper incisor, intact periodontium, 11,924 elements/20,852 nodes, Von Mises and maximum principal stress criteria, non-specified load) reported lower amounts of cervical stress (1.664 KPa for intrusion/extrusion, 0.6 KPa for translation, 0.54 KPa for tipping, and 0.015 KPa for rotation) for similar movements (intrusion/extrusion/tipping/translation/rotations) and VM criteria, as well as comparable stress areas. These differences are due to different loads, different tooth anatomies (incisor vs. premolar here), and less anatomical accuracy (507 fewer elements than our study).

Field et al. [14] in a numerical simulation (sample size of one) assessed tipping (0.35 N applied/0.5 N resulting) in two intact periodontium mandibular 3D models (first: 32,812 elements, with incisor, canine, and first premolar; second: 23,565 elements, canine; global element size of 1.2 mm, Von Mises, maximum and minimum principal stresses, and hydrostatic pressure), reporting higher stresses in a multi-teeth model when compared with a single-tooth one. In the trabecular/cancellous component stress distribution areas resembled here (alveolar socket concentrations), their intensity is unnatural (red—high stress, surrounding alveolar socket for both models, with a higher extent in multi-teeth models) for such a small force (0.35 N) when compared with our study (blue-green—small stress for 0.6–2.4 N, more natural display) and acknowledges clinical biomechanical behavior. If Field et al.’s [14] high red stresses were correct, the significance would be high ischemia and resorption for 0.35 N in an intact periodontium, which, in clinical reality, never occurs. The quantitative reports for the trabecular component were 34.2 KPa (single-tooth models) and 125.6 KPa (four times higher in multi-teeth models), which is also unrealistic (contradicting all biomechanical principles). Regarding the amount, ours were two times smaller (14.54 KPa, for 0.6 N) when compared with Field et al. (34.2 KPa, for 0.35 N). Moreover, they also reported amounts of 235.5–324.5 KPa in PDL (Von Mises criteria) in the same models, 15 times higher than the physiological 16–22 KPa of the MHP (meaning ischemia and resorption for 0.35 N intact periodontium models), which is deeply unrealistic [30]. These reports are potentially due to the reduced anatomical accuracy of the analyzed models (23,565–32,812 elements, global element size of 1.2 mm vs. 5.06–6.05 million elements, 0.97–1.07 million nodes, and a global element size of 0.08–0.116 mm here), modeling and boundary conditions issues, and lack of correlation with both MHP and available clinical data.

Merdji et al. [15] assessed three forces (10 N of intrusion and 3 N of tipping and translation) in an FEA simulation (Von Mises) with a sample size of one, in a lower-third molar intact periodontium model (142,305 elements, global element size of 0.25–1 mm), and reported a bone stress concentration in an intact periodontium in the cervical third (resembling our results). However, there are differences especially due to modeling and anatomical issues. These issues also influenced the reported stress amounts (extremely high: 10.5 MPa for 10 N intrusion, 11.5 MPa for 3 N tipping, and 16.83 MPa for 3 N translation vs. 40.7 KPa for 2.4 N intrusion, 58.17 KPa for 2.4 N tipping, and 79.25 KPa for 2.4 N translation here).

The limits of FEA studies are related to the fact that they cannot yet entirely reproduce clinical conditions. Further algorithms need to be created to properly reproduce the movements, clinical interactions between components, and internal tissular micro-architecture. However, since the currently available knowledge about FEA in dentistry is in its infancy (no numerical studies assessing the above-mentioned and providing solutions for making FEA as thrustable as in the engineering field), our study argued some of these on the basis of available data [4,5,6,11,21].

Our earlier studies reported that dental tissues are ductile-like materials with a certain brittle flow mode; thus, only ductile failure criteria (Tresca and Von Mises) could provide reliable results, with T being more correct than VM [1,2,17,20,22,23]. Moreover, up to 2.4 N, the assumed boundary conditions (isotropy, linear elasticity, and non-homogeneity/homogeneity) are correct [1,17,20], as reported here. Isotropy, linear elasticity, and homogeneity assumptions were also used in most of the earlier dental FEA studies [4,5,6,7,8,9,10,11,12,13,14,15,21], despite the anatomical reality of anisotropy, non-homogeneity, and nonlinear elasticity [1,2,17,22].

In our study, the non-homogeneity nature was addressed by the selection of the Tresca failure criterion, specially designed for non-homogenous materials. The linear elasticity assumption is biomechanically correct only for small loads, where there are extremely small displacements and deformations [1,2,17].

The anisotropy–isotropy issue is still a subject of controversy since there are contradictory reports, being studied only for the PDL [27,28,29]. Hemanth et al. [27,28] reported nonlinear quantitative results to be 20–50% less than the linear results (0.3–1 N for intrusion and tipping). However, the main issue related to Hemanth et al.’s [27,28] reports were that the PDL (of upper incisor) was assessed as a brittle-like material employing maximum principal stress (despite clinically being ductile-like, fundamentally changing the studied biomechanics) under forces less than 1 N (despite classical mechanics knowledge reporting that all materials show linear elasticity for that range). By contrast, Toms et al. [29] reported higher quantitative values for the PDL of the lower premolar (under 1 N of extrusion) for the nonlinear assumption when compared with the linear assumption. However, despite using Von Mises criteria, Toms et al. [29] reported unrealistic stress distribution areas (qualitative results), contradicting the available clinical knowledge.

As part of boundary conditions, our analyses applied a uniform loaded area (linear function) with a ramp amplitude (small increments up to entire load) to prevent stress concentrations, premature failure, and numerical problems. No mentions about the loading conditions were found in the above-mentioned FEA studies; thus, the differences between our results and theirs could also be related to the FEA loading phase.

The sample size of FEA analyses is generally accepted to be one [4,5,6,7,8,9,10,11,12,13,14,15,16,21,24,27,28,29], since numerical analyses are descriptive studies that are fundamentally different from clinical ones [1,17,22]. FEA studies, due to the multiple possibilities of changing study conditions, can produce many studies with fundamentally different results by analyzing only a single model. However, the accuracy of this study is increased if the sample size is larger. Thus, our analyses used a sample size of nine (nine patients, 81 models, and 405 simulations).

Despite all the above-mentioned limitations, FEA is the only method allowing the individual study of tissular dental components and providing results and conclusions applicable in both clinical practice and scientific research. Our study aimed to assess both the biomechanical behavior of living periodontal structures with various levels of bone loss (0–8 mm) under multiple orthodontic movements and the ischemic and resorptive risks present at the tissular level with high impact during the orthodontic phase. To achieve this, we needed numerical models of high anatomical accuracy possessing the natural geometrical tissular architecture of the living structures. Thus, the 3D models based on X-ray CBCT examination seemed the best choice. Since the natural periodontal living tissues have anisotropy, non-homogeneity, and nonlinear elasticity as physical properties, we needed our failure criteria to be suitable for our 3D models. Tresca criteria were the only ones to meet all the above-mentioned requirements. However, their results need to be correlated with clinical data and available knowledge to verify their accuracy.

## 5. Conclusions

1. Both failure criteria showed similar qualitative results, while the quantitative results were 1.09–1.21 higher for T when compared with VM. There were no qualitative differences between the stress resulting from the three orthodontic loads. Quantitatively, a doubling was seen for 1.2 N and quadrupling for 2.4 N when compared to 0.6 N.
2. Rotation and translation followed by tipping seemed to be the most stressful movements especially for a reduced periodontium, prone to higher ischemic and resorptive risks.
3. If, in an intact periodontium with up to 1.2 N, there seemed to be no major ischemic and resorptive risks, in a reduced periodontium, 1.2 N could be used only for extrusion and intrusion. For the other three movements, more than 0.6 N is prone to increasing ischemic and resorptive risks.
4. Biomechanically, in an intact periodontium, the stress is spread in the alveolar socket and entire trabecular structure. In a reduced periodontium, a visible concentration and increase in stress around the cervical third of the remaining alveolar socket after 4 mm of loss is visible, for all orthodontic movements and loads.
5. Biomechanically, it seems that 4 mm of loss is a marker for the change in stress distribution during periodontal breakdown. A visible correlation between bone loss progress, changes in stress distribution (especially concentrations around the alveolar socket after 4 mm), and quantitative stress increase was seen.

### Practical Implications

In clinical practice, patients with various levels of periodontal breakdown are relatively common; thus, knowing the changes produced in biomechanical behavior by bone loss is of extreme importance. The amount of applied orthodontic forces is still a subject of controversy for an intact periodontium, while for a reduced periodontium, less data are available. Thus, knowing that up to 1.2 N can be safely applied in an intact periodontium and more than 0.6 N should be considered with care for a reduced periodontium are of extreme importance in minimizing ischemic and resorptive risks. Moreover, knowing that, in an intact periodontium, stress is spread both around the alveolar socket and entire trabecular structure while that after 4 mm of loss tends to concentrate around alveolar socket is also important for a practitioner. This study enhances the knowledge about the trabecular bone behavior during periodontal breakdown.

In scientific research, since there are no data about the trabecular bone component, a study that provides a clear and correct picture of the biomechanical behavior of both an intact and reduced periodontium under increasing orthodontic forces and movements is valuable. Moreover, it is rare to provide an FEA study that produces correct results that are in accordance with both physiological constants and clinical data. This study helps the researcher by completing the general image of the failure criteria, boundary conditions and assumptions, and method, to better understand and to effectively use the FEA method, obtaining correct dental studies in engineering field.

## Figures and Tables

**Table 1:** Elastic properties of materials.

Material	Young’s Modulus, E (GPa)	Poisson Ratio, ʋ	Refs.
Enamel	80	0.33	[1,2,17,20,22,23]
Dentin/Cementum	18.6	0.31	[1,2,17,20,22,23]
Pulp	0.0021	0.45	[1,2,17,20,22,23]
PDL	0.0667	0.49	[1,2,17,20,22,23]
Cortical bone	14.5	0.323	[1,2,17,20,22,23]
Trabecular bone	1.37	0.3	[1,2,17,20,22,23]
Stainless Steel	190	0.265	[1,2,17,20,22,23]

**Table 2:** Maximum stress average values (KPa) produced by the three orthodontic loads/forces.

Resorption (mm)			0	1	2	3	4	5	6	7	8
Intrusion	Tresca	a	4.13	4.20	4.27	4.34	4.42	5.16	5.90	6.64	7.38
0.6 N/60 gf		m	8.31	8.97	9.64	10.30	10.97	12.86	14.75	16.64	18.43
		c	11.07	13.23	15.39	17.55	19.72	23.07	26.43	29.78	33.14
	VM	a	2.97	3.19	3.41	3.64	3.86	4.51	5.15	5.80	6.44
		m	7.28	7.87	8.44	9.02	9.61	11.22	12.84	14.46	16.08
		c	10.18	11.95	13.72	15.49	17.26	20.17	23.09	26.00	28.92
1.2 N/120 gf	Tresca	a	8.26	8.40	8.54	8.68	8.83	10.32	11.80	13.28	14.77
		m	16.63	17.94	19.28	20.60	21.95	25.72	29.50	33.28	36.86
		c	22.14	26.46	30.78	35.10	39.45	46.14	52.86	59.56	66.28
	VM	a	5.94	6.38	6.83	7.27	7.73	9.01	10.30	11.59	12.89
		m	14.56	15.73	16.88	18.04	19.21	22.44	25.68	28.92	32.17
		c	20.36	23.90	27.44	30.98	34.52	40.34	46.18	52.00	57.85
2.4 N/240 gf	Tresca	a	16.52	16.80	17.08	17.36	17.66	20.64	23.60	26.56	29.53
		m	33.25	35.88	38.56	41.20	43.89	51.44	59.00	66.56	73.72
		c	44.29	52.92	61.56	70.20	78.89	92.28	105.72	119.12	132.57
	VM	a	11.88	12.77	13.66	14.55	15.46	18.02	20.60	23.18	25.77
		m	29.12	31.46	33.76	36.08	38.43	44.88	51.36	57.84	64.34
		c	40.73	47.80	54.88	61.96	69.05	80.68	92.36	104.00	115.69
Extrusion	Tresca	a	4.13	4.20	4.27	4.34	4.42	5.16	5.90	6.64	7.38
0.6 N/60 gf		m	8.31	8.97	9.64	10.30	10.97	12.86	14.75	16.64	18.43
		c	11.07	13.23	15.39	17.55	19.72	23.07	26.43	29.78	33.14
	VM	a	2.97	3.19	3.41	3.64	3.86	4.51	5.15	5.80	6.44
		m	7.28	7.87	8.44	9.02	9.61	11.22	12.84	14.46	16.08
		c	10.18	11.95	13.72	15.49	17.26	20.17	23.09	26.00	28.92
1.2 N/120 gf	Tresca	a	8.26	8.40	8.54	8.68	8.83	10.32	11.80	13.28	14.77
		m	16.63	17.94	19.28	20.60	21.95	25.72	29.50	33.28	36.86
		c	22.14	26.46	30.78	35.10	39.45	46.14	52.86	59.56	66.28
	VM	a	5.94	6.38	6.83	7.27	7.73	9.01	10.30	11.59	12.89
		m	14.56	15.73	16.88	18.04	19.21	22.44	25.68	28.92	32.17
		c	20.36	23.90	27.44	30.98	34.52	40.34	46.18	52.00	57.85
2.4 N/240 gf	Tresca	a	16.52	16.80	17.08	17.36	17.66	20.64	23.60	26.56	29.53
		m	33.25	35.88	38.56	41.20	43.89	51.44	59.00	66.56	73.72
		c	44.29	52.92	61.56	70.20	78.89	92.28	105.72	119.12	132.57
	VM	a	11.88	12.77	13.66	14.55	15.46	18.02	20.60	23.18	25.77
		m	29.12	31.46	33.76	36.08	38.43	44.88	51.36	57.84	64.34
		c	40.73	47.80	54.88	61.96	69.05	80.68	92.36	104.00	115.69
Translation	Tresca	a	8.06	8.26	8.46	8.66	8.87	9.88	10.90	11.92	12.94
0.6 N/60 gf		m	17.08	19.14	21.20	23.26	25.32	35.08	44.85	54.61	64.38
		c	22.74	28.65	34.56	40.47	46.38	63.75	81.12	98.49	115.87
	VM	a	7.05	7.24	7.43	7.62	7.81	8.68	9.55	10.42	11.30
		m	14.88	16.91	18.95	20.98	23.02	31.31	39.60	47.89	56.18
		c	19.81	25.39	30.98	36.56	42.15	56.89	71.63	86.37	101.12
1.2 N/120 gf	Tresca	a	16.12	16.52	16.92	17.32	17.75	19.76	21.80	23.84	25.89
		m	34.17	38.28	42.40	46.52	50.64	70.16	89.70	109.22	128.76
		c	45.48	57.30	69.12	80.94	92.75	127.50	162.24	196.98	231.74
	VM	a	14.10	14.48	14.86	15.24	15.61	17.36	19.10	20.84	22.60
		m	29.76	33.82	37.90	41.96	46.04	62.62	79.20	95.78	112.36
		c	39.63	50.78	61.96	73.12	84.31	113.78	143.26	172.74	202.25
2.4 N/240 gf	Tresca	a	32.23	33.04	33.84	34.64	35.50	39.52	43.60	47.68	51.77
		m	68.34	76.56	84.80	93.04	101.28	140.32	179.40	218.44	257.53
		c	90.97	114.60	138.24	161.88	185.50	255.00	324.48	393.96	463.48
	VM	a	28.19	28.96	29.72	30.48	31.23	34.72	38.20	41.68	45.21
		m	59.53	67.64	75.80	83.92	92.08	125.24	158.40	191.56	224.72
		c	79.25	101.56	123.92	146.24	168.62	227.56	286.52	345.48	404.50
Rotation	Tresca	a	11.56	13.78	16.00	18.22	20.45	20.87	21.30	21.72	22.15
0.6 N/60 gf		m	22.86	24.79	26.72	28.65	30.59	39.39	48.20	57.01	65.82
		c	22.86	37.47	52.00	66.71	81.33	91.12	100.91	110.70	120.50
	VM	a	10.51	12.34	14.17	16.00	17.84	18.18	18.52	18.86	19.21
		m	20.76	22.23	23.70	25.17	26.65	34.26	41.88	49.50	57.12
		c	20.76	33.27	45.78	58.29	70.81	79.24	87.67	96.10	104.53
1.2 N/120 gf	Tresca	a	23.13	27.56	32.00	36.44	40.91	41.74	42.60	43.44	44.29
		m	45.71	49.58	53.44	57.30	61.19	78.78	96.40	114.02	131.64
		c	45.71	74.94	104.00	133.42	162.65	182.24	201.82	221.40	240.99
	VM	a	21.01	24.68	28.34	32.00	35.69	36.36	37.04	37.72	38.42
		m	41.52	44.46	47.40	50.34	53.30	68.52	83.76	99.00	114.24
		c	41.52	66.54	91.56	116.58	141.62	158.48	175.34	192.20	209.06
2.4 N/240 gf	Tresca	a	46.26	55.12	64.00	72.88	81.81	83.48	85.20	86.88	88.58
		m	91.42	99.16	106.88	114.60	122.37	157.56	192.80	228.04	263.28
		c	91.42	149.88	208.00	266.84	325.31	364.48	403.64	442.80	481.98
	VM	a	42.02	49.36	56.68	64.00	71.37	72.72	74.08	75.44	76.84
		m	83.05	88.92	94.80	100.68	106.59	137.04	167.52	198.00	228.48
		c	83.05	133.08	183.12	233.16	283.24	316.96	350.68	384.40	418.12
Tipping	Tresca	a	6.20	6.54	6.88	7.22	7.57	9.39	11.21	13.03	14.86
0.6 N/60 gf		m	15.15	16.92	18.70	20.48	22.26	31.45	40.65	49.84	59.04
		c	15.15	22.44	29.73	37.02	44.31	59.05	73.79	88.53	103.28
	VM	a	5.95	6.11	6.27	6.43	6.60	8.19	9.79	11.38	12.98
		m	14.54	15.75	16.96	18.17	19.39	2.44	35.50	43.56	51.62
		c	14.54	20.54	26.55	32.56	38.57	51.48	64.40	77.31	90.23
1.2 N/120 gf	Tresca	a	12.40	13.08	13.76	14.44	15.14	18.78	22.42	26.06	29.71
		m	30.31	33.84	37.40	40.96	44.52	62.90	81.30	99.68	118.09
		c	30.31	44.88	59.46	74.04	88.61	118.10	147.58	177.06	206.56
	VM	a	11.90	12.22	12.54	12.86	13.20	16.38	19.58	22.76	25.96
		m	29.08	31.50	33.92	36.34	38.77	4.88	71.00	87.12	103.24
		c	29.08	41.08	53.10	65.12	77.13	102.96	128.80	154.62	180.46
2.4 N/240 gf	Tresca	a	24.80	26.16	27.52	28.88	30.29	37.56	44.84	52.12	59.42
		m	60.62	67.68	74.80	81.92	89.05	125.80	162.60	199.36	236.17
		c	60.62	89.76	118.92	148.08	177.23	236.20	295.16	354.12	413.11
	VM	a	23.80	24.44	25.08	25.72	26.39	32.76	39.16	45.52	51.92
		m	58.17	63.00	67.84	72.68	77.55	9.76	142.00	174.24	206.47
		c	58.17	82.16	106.20	130.24	154.27	205.92	257.60	309.24	360.93

a—apical third, m—middle third, c—cervical third.

## Data Availability

All necessary data is in the manuscript.
